# Using cultural historical activity theory to reflect on the sociocultural complexities in OSCE examiners’ judgements

**DOI:** 10.1007/s10459-022-10139-1

**Published:** 2022-08-09

**Authors:** Wai Yee Amy Wong, Jill Thistlethwaite, Karen Moni, Chris Roberts

**Affiliations:** 1 School of Education and Faculty of Medicine, The University of Queensland, Brisbane, QLD 4072 Australia; 2 Faculty of Health, The University of Technology Sydney, Sydney, NSW 2007 Australia; 3 School of Education, The University of Queensland, Brisbane, QLD 4072 Australia; 4 Sydney Medical School, Faculty of Medicine and Health, The University of Sydney, Sydney, NSW 2006 Australia; 5 School of Nursing and Midwifery, Queen’s University Belfast, Belfast, BT9 7BL UK

**Keywords:** Cultural historical activity theory, Assessment, Faculty development, Feedback, Judgement, OSCE

## Abstract

Examiners’ judgements play a critical role in competency-based assessments such as objective structured clinical examinations (OSCEs). The standardised nature of OSCEs and their alignment with regulatory accountability assure their wide use as high-stakes assessment in medical education. Research into examiner behaviours has predominantly explored the desirable psychometric characteristics of OSCEs, or investigated examiners’ judgements from a cognitive rather than a sociocultural perspective. This study applies cultural historical activity theory (CHAT) to address this gap in exploring examiners’ judgements in a high-stakes OSCE. Based on the idea that OSCE examiners’ judgements are socially constructed and mediated by their clinical roles, the objective was to explore the sociocultural factors that influenced examiners’ judgements of student competence and use the findings to inform examiner training to enhance assessment practice. Seventeen semi-structured interviews were conducted with examiners who assessed medical student competence in progressing to the next stage of training in a large-scale OSCE at one Australian university. The initial thematic analysis provided a basis for applying CHAT iteratively to explore the sociocultural factors and, specifically, the contradictions created by interactions between different elements such as examiners and rules, thus highlighting the factors influencing examiners’ judgements. The findings indicated four key factors that influenced examiners’ judgements: examiners’ contrasting beliefs about the purpose of the OSCE; their varying perceptions of the marking criteria; divergent expectations of student competence; and idiosyncratic judgement practices. These factors were interrelated with the activity systems of the medical school’s assessment practices and the examiners’ clinical work contexts. Contradictions were identified through the guiding principles of multi-voicedness and historicity. The exploration of the sociocultural factors that may influence the consistency of examiners’ judgements was facilitated by applying CHAT as an analytical framework. Reflecting upon these factors at organisational and system levels generated insights for creating fit-for-purpose examiner training to enhance assessment practice.

**Supplementary Information:**

The online version contains supplementary material available at 10.1007/s10459-022-10139-1.

## Introduction

The objective structured clinical examination (OSCE) has been widely adopted in medical education as a competency-based assessment (CBA) for assessing medical students’ clinical practice (Khan et al., 2013; Wong et al., 2020). The perceived objectivity of the OSCE (Harden et al., 2015; Wong et al., 2020) and its alignment with the regulatory accountability for professional accreditation accounts for its dominance in clinical assessment (Reid et al., 2021). In an OSCE, students are presented with standardised medical problems in a series of stations. Examiners are given marking criteria in the form of standardised checklists, domain-based ratings or behaviourally anchored rating scales (BARS) (Homer et al., 2020). There is a fundamental research issue as to whether examiner judging behaviours can be truly objective when assessing students in practice, and amenable to intervention through, for example, training. If examiner behaviours are subjective, this would require a different approach to researching examiner behaviours and inferring appropriate training interventions. Research into examiner behaviours has predominantly explored the desirable psychometric characteristics of OSCEs (Bartman et al., 2013; Fuller et al., 2017; Harasym et al., 2008; McManus et al., 2006), or investigated examiners’ judgements from a cognitive perspective (Gingerich et al., 2014; Yeates et al., 2013, 2019) rather than from a sociocultural perspective. This presents as a gap in the research which is the focus of this paper.

Within the standardised approach, OSCE designers need to ensure the objectivity and reliability of examiners’ judgements, particularly in summative assessments judged by a sole examiner (Berendonk et al., 2013), in order to make high-stakes decisions about student progression to the next stage of their medical training. Individual examiners’ subjectivity and consequent divergence from the standardised marking criteria are thought to compromise the perceived objectivity of ratings of student performance in an OSCE (Bartman et al., 2013; Harasym et al., 2008; McManus et al., 2006; Williams et al., 2003; Yeates et al., 2013). Examiner training has been introduced in an attempt to decrease variations in examiners’ judgements (Cook et al., 2009; Holmboe et al., 2004; Malau-Aduli et al., 2012; Pell et al., 2008). However, results of these examiner training studies have been inconclusive and difficult to compare as researchers applied different methodologies (Reid et al., 2016).

Scholars have also drawn attention to limitations in the standardised psychometric approach to OSCE design, advocating for new approaches to understanding examiner behaviours and training. Ten Cate and Regehr (2019), for example, argued that examiners’ judgements of clinical competence are inherently subjective and heavily dependent on context. From these perspectives, OSCEs are situated in a complex social and cultural context in which examiners interact with students in a constructed socio-clinical scenario (Gormley et al., 2021). Examiners can perceive the same student performance differently based on legitimate reasons influenced by their clinical experience (Ten Cate & Regehr, 2019).

Developing a deeper understanding of the sociocultural factors that influence examiners’ judgements with a systemic and multi-dimensional approach could inform the design of effective, fit-for-purpose OSCE examiner training that embraces examiners’ legitimate subjectivity of their judgements. Such an approach would also align with the mutually inclusive perspectives of examiner cognition research which recognise examiners as trainable, fallible, or meaningfully idiosyncratic (Gingerich et al., 2014). The study reported here focused on a large final-year medical OSCE in one Australian research-intensive university to identify and explore the sociocultural factors that influenced examiners’ judgements of student competence.

### Analytical framework: cultural historical activity theory (CHAT)

Cultural Historical Activity Theory (CHAT) (Engeström, 2001, 2004, 2014) has afforded the research community an innovative theoretical lens to qualitatively explore the research phenomena of examiner behaviours in a complex context. CHAT has been applied previously in medical education research (Cleland et al., 2016; Kajamaa et al., 2019; Larsen et al., 2017; Morris, 2012), but not specifically to explore the practice of examiners’ judgements within student assessment. To the best of our knowledge, this is the first empirical qualitative study using CHAT as an analytical framework in medical education assessment research. CHAT presented a systemic and multi-dimensional approach to exploring a comprehensive set of dynamic factors (Foot, 2014) such as culture, history and examiners’ social interactions that may influence examiners’ judgements of student competence.

**Fig. 1:**
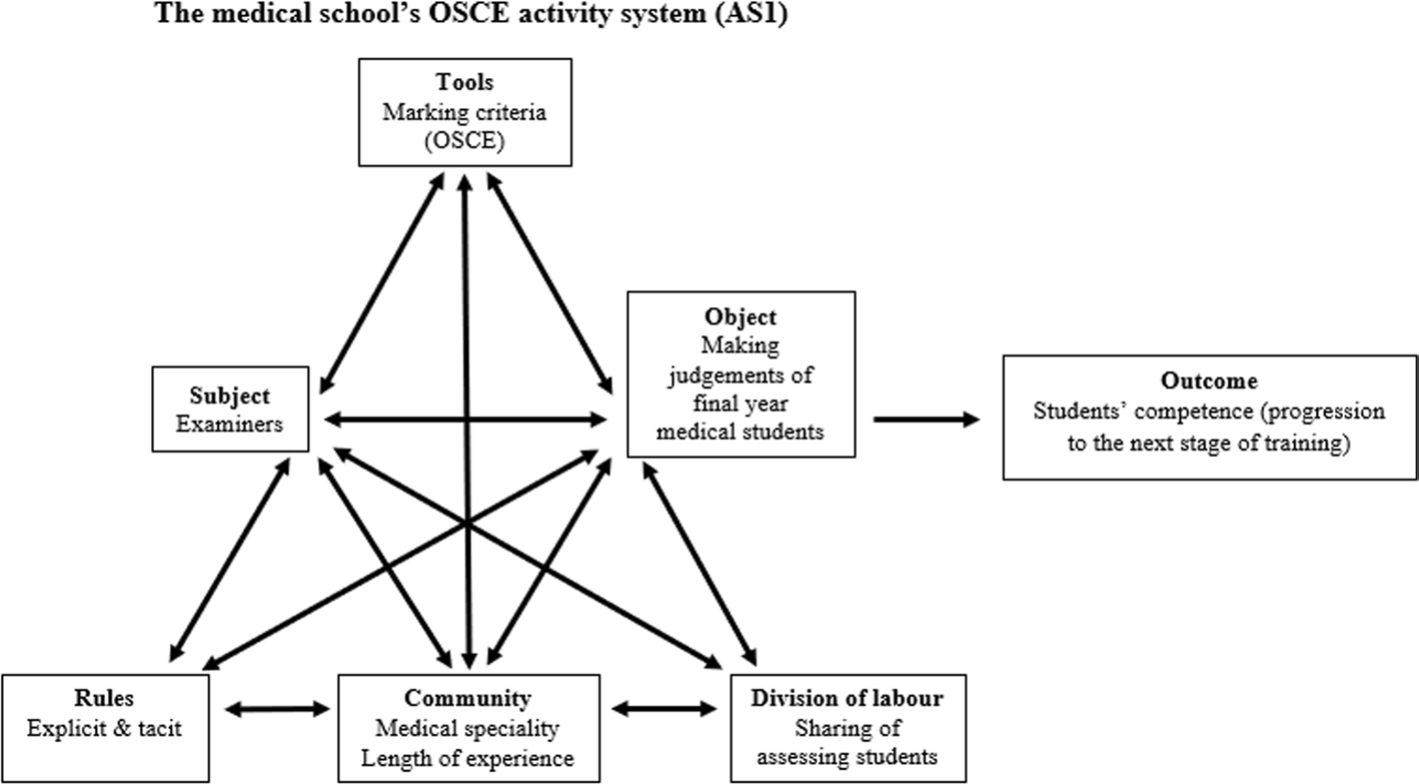
The medical school’s OSCE activity system (AS1). Adapted from Engeström (2014)

CHAT has also provided a robust framework to analyse professional work practices (Foot, 2014), which in the current study refers to the judgement practices of the OSCE examiners (subject) in an activity system (the medical school). Each activity system is a basic unit of analysis which consists of six elements: subject, object, tools, rules, community, and division of labour (Engeström, 2014). The primary activity system (AS1) in this study is the medical school’s OSCE activity system (Fig. 1). It is made up of OSCE station marking criteria (tools) which mediate the OSCE examiner’s (subject) actions to fulfil the goal (object) of making judgements. Further details of each of the elements in AS1 are outlined in Table 1.

**Table 1:** Elements in the primary activity system (AS1) within the medical school OSCE context

Elements	Definitions	Relevance to this Study
A subject	Refers to people who collectively engage in the activity (Engeström, 2014)	The examiners who assessed medical students in their final-year OSCE
An object	Describes the goal of the activity (Lazarev, 2004; Leont'ev, 1974)	The goal was to make judgements of the clinical competence of final-year medical students
Tools	Contributes to the experience of the subject (Cole & Engeström, 1993)	The focus of the tools was the OSCE station marking criteria
Rules	Explicit rules	These rules specify collective conventions (Bligh & Flood, 2015) used in this medical school, and formal regulations (Engeström, 2014) mandated by the Australian Medical Council (AMC) which defines standards and accredits medical programmes in Australia
	Tacit rules	These rules represent the codes of interactions (Roth & Lee, 2007) among the OSCE examiners and the assumed rules set by the medical school which could be learnt through examiner training or experience
A community	Refers to the social group that a person or groups of people (subject) belong to (Engeström, 2014)	The examiners were groups of people from different medical specialties. They played different roles within the medical school depending on their length of experience Apart from the examiners, there were three other social groups: the medical school, the final-year medical students, and simulated patients
Division of labour	Describes the sharing of tasks among groups of people (subject) (Engeström, 2014) Defines the assignment of responsibilities based on the subject’s areas of expertise and hierarchy (Bligh & Flood, 2015)	The examiners shared the tasks of assessing all the final-year medical students within two days The examiners played different roles and were assigned with different responsibilities. For example, the experienced examiners were more likely to be the chief examiner of an OSCE station. The chief examiner conducted the briefing before each OSCE session The final-year medical students had the responsibilities to complete the OSCE The simulated patients had the responsibilities to act according to the given instructions in an OSCE station
An outcome	Represents the end results of the object-oriented activity (Engeström, 2014)	To assess medical students’ competence for progression to the next stage of clinical training

On a conceptual level, interactions among the elements of rules, community and division of labour are expected to support the examiners to achieve the desired outcome of assessing medical students’ competence for progression to the next stage of clinical training. However, multiple points of view, traditions and interests of examiners based on their own diverse histories (Engeström, 2018) have the potential to create contradictions within an activity system which impact on achieving the desired outcome (Engeström, 2014). In addition, different elements within AS1 relate to each other and are continuously influencing and being influenced by others and hence are illustrated by the double-sided arrows between each element (Al-Ali, 2020) (Fig. 1).

The OSCE examiners were also medical practitioners working in the clinical environment which is the second activity system (AS2) in this study. It is possible to have multiple activity systems (the medical school and the clinical environment) interacting with each other to achieve shared outcomes or create contradictions (Engeström, 2014). The examiners might also feel obliged to make judgements of the final-year medical students undertaking the OSCE as to whether they could be safe and competent interns, thus creating further contradictions within and across AS1 and AS2. The guiding principles of CHAT such as multi-voicedness (e.g., multiple voices from the examiners and the medical school), and historicity (e.g., local history embedded in the rules and systems) (Engeström, 2001, 2004) also help identify the contradictions through exploring the interactions among different elements in the activity systems. Understanding these contradictions is essential to developing a deeper understanding of the complexity of the sociocultural factors that influence examiners’ judgements of student performance in high-stakes OSCEs, hence making more situated recommendations for examiner training initiatives.

The aim of this study was to address the research question ‘What are the sociocultural factors that influenced examiners’ judgements of student competence through the theoretical lens of CHAT?’ The key objective was to develop a theoretically informed explanation of examiner behaviours with a view to informing the development of training to enhance examiner practices.

## Methods

### Contextual background

This paper reports the qualitative component of a mixed-methods doctoral case study exploring the consistency of examiners’ judgements in a high-stakes OSCE at the medical school of an Australian research-intensive university (Wong, 2019), one of the largest in the country. There has been no national licensing examination to become a medical practitioner in Australia. Each medical school develops its own final examinations based on the curriculum accredited by the Australian Medical Council (AMC) for the Medical Board of Australia. The OSCE in this study was the final examination conducted at the end of the entire graduate entry four-year medical programme. The results of this high-stakes OSCE had a direct impact on decision making about student progression to the conferral of their medical degree and into internships.

Among the examiners, there was a range of experience in terms of assessing students in their end-of-programme OSCE, which created additional challenges in ensuring consistency of their judgements and implementing homogenous examiner training. Every year over 100 volunteer examiners, who were or had been practising clinicians from a range of specialties, were involved. Some of these clinicians regularly engaged with teaching medical students, whereas the majority only interact with students during the annual final-year OSCE. At the time of the study, the examiners assessed more than 350 medical students in four sessions on two consecutive days across four sites. A single examiner was allocated to each station which is a common practice in large-scale OSCEs (Roberts et al., 2006), which continues in our experience, thus demonstrating the relevancy of the study’s findings to current OSCEs.

**Fig. 2:**
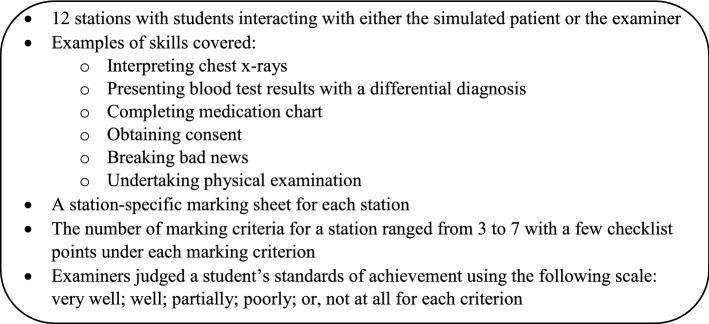
Details of the OSCE stations and marking criteria

All examiners received written information about their role in the OSCE and attended a short briefing (around 30 min) before each session led by an experienced examiner. The examiners were required to score student performance on the station-specific marking criteria sheet, which had been developed and refined over time by the final-year OSCE design team consisting of clinicians and medical educators at the medical school. There were three parts to score for each station. Part A was criterion-referenced scoring comprising a list of relevant criteria to assess a specific clinical skill, or clinical scenario. Examiners were required to score each student’s performance based on the following marking standards: *very well; well; partially; poorly;* or*, not at all*. Part B asked for the examiner’s global impression of a student’s performance in a station. This part was identical for all stations and examiners were required to assess each student based on the following marking standards: *pass; borderline; fail* or *unacceptable/unsafe*. Part C of the marking sheet asked the examiners to provide comments, particularly for the borderline or fail students. Additional details of the OSCE stations and marking criteria are outlined in Fig. 2.

### Participants

The lead author recruited examiners to participate in the research, based on a convenience sample of those wishing to participate from each of the four sites that conducted the OSCE. All examiners, who assessed the final-year medical students when this study was conducted, received an invitation email to participate in this study.

### Ethical statement

This study was approved by the University’s Behavioural & Social Sciences Ethical Review Committee (Approval No: 2013001070). All participants were provided with a participant information sheet which stated that their participation was voluntary and did not have any implications for their employment with the university. They provided informed consent through an online link or replying to the invitation email. All participants were asked for verbal consent prior to commencing the audio-recording of the interviews. The lead author’s university approved the arrangement with the transcription company.

### Data collection

Qualitative data were collected by the lead author through semi-structured interviews with 17 OSCE examiners at a place and time convenient to them; these lasted an average of 17 min. The interviews were conducted as a conversation around their general beliefs and experiences as OSCE examiners based on the interview guide (Online Appendix 1) which was developed from the insights of the quantitative findings of this mixed-methods study (Wong et al., 2020). The interviews allowed examiners to elaborate on their perceptions of the major challenges in making judgements of student competence in the OSCE, their interpretations of subjectivity, and specific factors that might influence their judgements. Although the number of interviews undertaken was dependent on the availability and willingness of the examiners, the lead author employed the Comparative Method for Themes Saturation (CoMeTS) (Constantinou et al., 2017) to compare the factors developed from each interview to ensure that saturation of data had been achieved. The lead author checked the accuracy of the transcriptions of the audio-recorded interviews prior to data analysis.

### Data analysis

Thematic analysis has been employed in activity system research (de Feijter et al., 2011; Lingard et al., 2012; Toth-Cohen, 2008). The lead author led the initial thematic analysis to analyse the interviews using open coding (Merriam & Tisdell, 2016). An interpretive paradigm was adopted (Allen & Jensen, 1996; Bunniss & Kelly, 2010; Weaver & Olson, 2006) focusing on exploring the sociocultural factors that influenced the examiners’ judgements. The open codes represented the examiners’ perceptions of their judgement behaviour. All the open codes were classified into different groupings using axial coding (Corbin & Strauss, 2015) and the recurring patterns of the groupings were analysed to consolidate them into a set of factors (Merriam & Tisdell, 2016). CHAT was then applied iteratively as a theoretical lens to consider the sociocultural contexts situated around these factors guided by the principles of multi-voicedness and historicity (Engeström, 2001, 2004). The primary activity system was based around the assessment practice in the medical school OSCE context (AS1) and the secondary was the examiners’ clinical work context (AS2). The examiners who assessed the final-year students in the medical school were also employed as medical doctors working in a clinical environment. Therefore, it was important to explore the interactions among the elements within and between these two distinct activity systems (AS1 and AS2) as to how they created contradictions and impacted on the examiners’ judgements.

### Trustworthiness

The lead author’s experience of conducting interviews gained during postgraduate studies, guidance provided by doctoral supervisors who are experienced researchers in higher and medical education, and the use of the interview guide enhanced the trustworthiness of the interview data collected. The lead author, as an insider-observer working closely with some of the examiners at the time when this study was conducted, always noted any potential prejudices by reflecting on the interpretation of the data and discussing these with the research team. The interdisciplinary research team with both JT and CR as medical doctors, experienced OSCE examiners and health professions educators and researchers, and KM as an experienced education researcher, enhanced the rigour of this study by engaging with CHAT from different perspectives. All authors engaged in the process of investigator triangulation (Merriam & Tisdell, 2016) through reading a proportion of the interviews independently and discussing the identified factors. They also contributed to applying CHAT as a framework of analysis and reflection to enhance the credibility and internal validity of this study.

## Findings

The analysis of the data identified four key sociocultural factors: examiners’ contrasting beliefs about the purpose of the OSCE; their varying perceptions of the marking criteria; divergent sociocultural expectations of student competence; and idiosyncratic judgement practices, that influenced examiners’ judgements across the contexts of the medical school and their clinical work. The findings are reported according to the sequence of analysis, that is, thematic followed by CHAT analysis. The findings indicated that the identified factors played a significant role in contributing to the sociocultural complexities of examiners’ judgements, and created contradictions, specifically in terms of multi-voicedness and historicity, in the activity systems.

1. *Examiners’ contrasting beliefs about the purpose of the end-of-programme OSCE*

Over half of the examiners believed the OSCE only assessed the clinical competence of students in a simulated environment implying that the demonstrated clinical competence may not be transferable to real-life clinical practice. They were concerned, therefore, that the OSCE mainly assessed the students’ ability to pass an examination. These examiners did not consider the end-of-programme OSCE as a fit-for-purpose hurdle for the final-year medical students to determine their competence for progression to internship and the next stage of training:

[… It is] an artificial situation, the exam. As you know, exams only test one thing, your ability to pass the exam, and they don't test anything else. (Examiner 5)

However, another group of six examiners believed that it was the last opportunity to ensure that only students who achieved the required clinical competence were awarded the opportunity to progress to internships. These examiners perceived that they played a gatekeeper role:

Overall, you have responsibility to the university and the community not just to let somebody through who perhaps has difficulties … something about their performance that might carry through if someone doesn’t pick it up and deal with it at the time. (Examiner 4)

Those examiners who believed that the OSCE mainly assessed student ability to pass the examination in an artificial environment were more likely to disregard the rules of not prompting students during the OSCE station as advised in the briefing meeting. They took into consideration that the students were undergraduates. In contrast, the examiners who believed that their role was gatekeeping were generally opposed to prompting students:

I guess if [students] need too much prompting, I think that they've misunderstood the station and they should fail. (Examiner 13)

CHAT was helpful in explaining the contradictions created by the examiners’ contrasting beliefs about the purpose of the end-of-programme OSCE. Focusing on the interactions of the examiners (subject) and the rules within the activity system (AS1), some of the examiners appeared to hold beliefs based on the explicit rules and considered themselves as gatekeepers. It is understandable in the context of this study with no national licensing examination that some examiners felt they were obliged to follow the medical school’s rules and the AMC regulatory requirements. Those examiners who perceived the end-of-programme OSCE as only assessing students’ ability to pass an examination were inclined to follow the tacit rule of providing students with guidance in an OSCE station. In addition, the fact that students were provided with opportunity to practise mock OSCEs prior to the actual OSCE could also influence the examiners’ belief. Students could be well-trained to undertake the end-of-programme OSCE so their performance might or might not be a genuine reflection of what they would do in the clinical context (AS2).

This misalignment between the examiners’ beliefs about the purpose of the end-of-programme OSCE and the medical school’s intended purpose as a final hurdle assessment generated contradictions among the community of examiners and individual examiners about the level of guidance that should be provided to students during the examination process. Different examiners’ distinct decisions about the extent of prompting or not prompting students during the OSCE illustrates the multi-voicedness within AS1. The contradictions created by these distinct decisions impacted on the examiners’ judgements of student competence.

2. *Examiners’ varying perceptions of usefulness of the OSCE marking criteria*

Marking criteria are the tools that assist examiners in making judgements. The examiners acknowledged that some aspects of the marking criteria were useful, while other aspects were less so. For example, the marking criteria helped the examiners construct a framework of meaning for their observations of student performance:

… I do use that [marking criteria] sheet to make sure that I'm on track with what's expected, but also if the student needs any help along the way, to guide them back to where they're supposed to be. (Examiner 13)

The majority of the examiners stated that safe practice was a critical criterion that had to be met in order for students to pass, regardless of the differences in their beliefs about the purpose of the end-of-programme OSCE. However, safe practice was not included as a specific marking criterion though examiners could indicate a student’s performance in a station was unsafe in the global impression mark (Part B). The absence of this critical criterion and the lack of clarity in the defined criteria for passing and failing students contributed to the examiners’ perceptions of the marking criteria as not being useful. Some examiners did not feel confident either to pass or fail students, and some diverged from the marking criteria when they judged student performance:

There was a marking sheet but I didn't feel there was a lot of guidance as to what was the expected level. What was a pass? What was a fail? So, I kind of had to make it up a bit on the spot. (Examiner 11)

This examiner’s response implied that the examiners in this end-of-programme OSCE did not share the same understanding of the marking standards and criteria.

Using CHAT as the framework of analysis and reflection, the examiners’ perceptions of the usefulness of the marking criteria were explored in the medical school OSCE activity system (AS1). These perceptions were associated with the interactions between the examiners (subject) and the OSCE marking criteria (tools) to make judgements of student competence (object). The interactions of the examiners with the marking criteria were explored through the principle of historicity. Some aspects of the marking criteria (tools) used at this medical school to address different rules of assessment had been stable such as using criterion-referenced marking. Some aspects had undergone significant changes, for example, failing students based on critical errors, informally referred to as ‘killer stations’ (Homer & Russell, 2021; Schuwirth & van der Vleuten, 2006).

My difficulty is that the marking systems are varied through the years … back to 1995 to 1997 … there was a heavy emphasis on criterion*-*referenced. So there were criteria laid out very explicitly and the expectation was that [students] would get the majority of those. That's where I developed my tendency to just tick off the things they'd done because it made it much easier to work out the mark … [The medical school] went through a phase where some of the stations had a designated critical error and if [students] either did or didn't do whatever it was then they failed that station no matter what … for me at times in the changeovers the transitions become a bit confusing. (Examiner 9)

Different examiners’ perceptions of the usefulness of the marking criteria also indicated that some examiners disagreed with the given marking criteria. The multi-voicedness in the examiners’ perceptions contributed to the contradictions among the examiner community when some of them diverged from the given marking criteria:

And if the marking criteria [do] not reflect sensibleness[sic], then I will diverge from that a little. (Examiner 3)

**Fig. 3:**
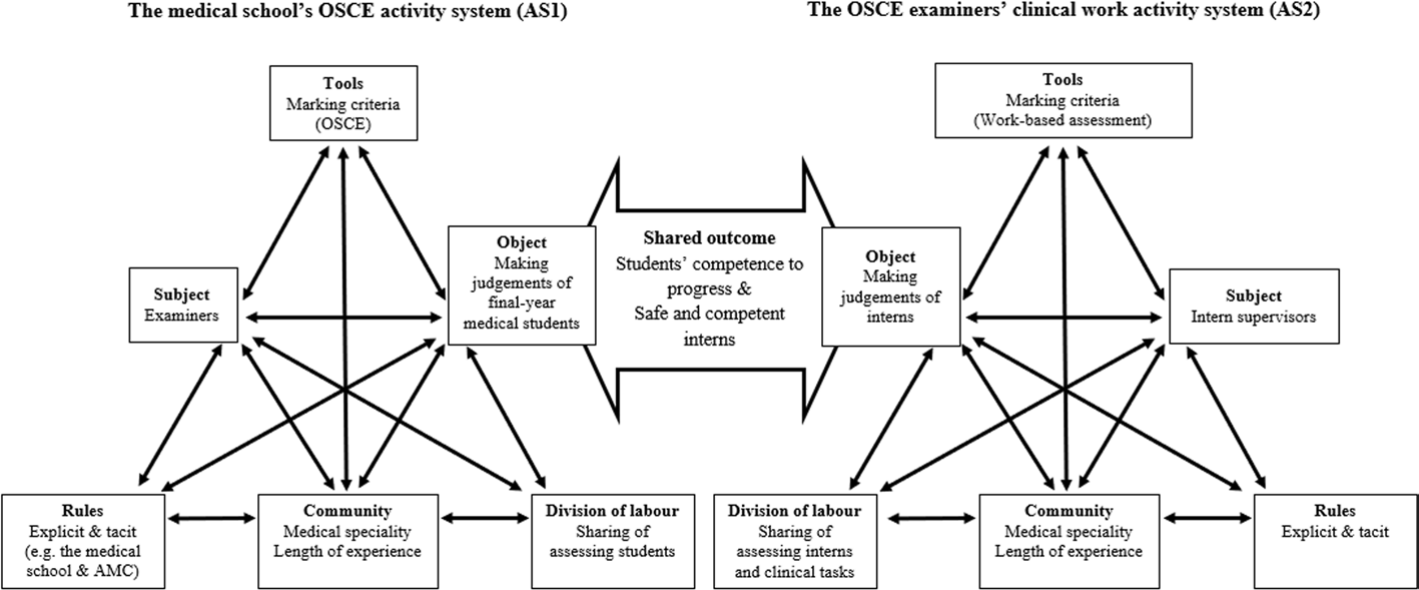
The medical school’s OSCE activity system (AS1) and the OSCE examiners’ clinical work activity system (AS2). Adapted from Engeström (2001)

In addition, the examiners’ perceptions of the marking criteria were mediated by the examiners’ clinical roles in their clinical work activity system (AS2) (Fig. 3). Some of the OSCE examiners in their everyday clinical roles were also intern supervisors. They employed a range of workplace-based assessments, for example, mini-clinical evaluation exercises (mini-CEXs), using different marking criteria (tools) to the OSCE, to assess their interns’ ongoing competence and identify their shortcomings for remediation purposes as mandated in the intern training. In this context, the examiners’ perceptions of the OSCE marking criteria were influenced by their experience of judging interns in their clinical work environment, as safe practice is a critical criterion for doctors performing clinical tasks in real-life situations:

It’s about whether the medical student can safely handle that as an intern. I just thought that the [marking criteria] wasn’t capturing that, the essence of the station. (Examiner 3)

The contradiction associated with the usefulness of the marking criteria is the fundamental distinction between safety and competence (Homer & Russell, 2021; Rushforth, 2007; Schuwirth & van der Vleuten, 2006). When using checklists for judging student performance in OSCE stations, students could pass the OSCE having demonstrated sufficient competence to obtain an overall pass mark, but not necessarily be safe to practise if this was not one of the specific checklist items. This is where the examiner’s global marking decision should be considered, as being safe to practise is critical in real-life situations.

The analysis of the interactions between AS1 and AS2 indicated that these two activity systems shared the outcome of assessing competence to determine the extent to which students and interns could be expected to be safe and competent in AS1 and AS2, respectively. However, the differences between assessing final-year medical students in a simulated environment and assessing interns in real-life clinical practice could create contradictions, specifically, when the examiners made judgements using the medical school’s marking criteria and diverged from them based on their experience with the interns in clinical settings. Therefore, the objectivity of examiners’ judgements became questionable as examiners applied the marking criteria differently.

3. *Examiners’ divergent sociocultural expectations of the final-year medical student competence*

Conceptualising OSCE as a complex sociocultural practice, the data analysis indicated that examiners’ expectations of final-year medical student competence were impacted by cultural, historical and social influences. From the cultural perspective, several social groups within the community of examiners in AS1 were identified based on the examiners’ medical specialties, for example, general practice or surgery. The examiners from different medical specialties had diverse expectations or a different focus in respect to the important aspects of student competence:

Being a GP [general practitioner], my whole job is based upon talking to people. If [students] strike me as the sort of person that can talk to people easily and translate medical ideas into easy language to understand for patients then I'm going to give them a good mark or I'm going to go softer [on them]. (Examiner 16)

The years of experience of being OSCE examiners were also associated with their expectations. The experienced examiners were assigned the responsibility as chief examiners who conducted the briefing before each OSCE session. They were entrusted with more power to lead the discussion and make final decisions on what was expected of students and examiners. This briefing with written resources was the only examiner training provided. The less-experienced examiners had less power and were inclined to follow the decisions made by the experienced OSCE examiners:

… it was a hernia exam essentially and there was an old retired surgeon … he was supervising and he said that he saw [the OSCE] more of a teaching exercise. That he didn't fail anyone … and that would have influenced me because I thought, this guy is an expert in the area … I was probably more guided by what he said. (Examiner 13)

The social implication of incompetent interns is another significant influence on the examiners’ expectations. The two most evident social expectations valued by these examiners were safe practice and effective communication with patients in real-life situations. The examiners indicated that graduating students should be able to safely perform the required clinical tasks, communicate effectively and develop trust with patients. These expectations were interrelated with the examiners’ belief about their role as gatekeepers. The OSCE examiners expected that the final-year students should have already developed a solid foundation of medical knowledge and professional behaviour on which to build their clinical experience in the intern year:

… I'm prepared to have you as one of my junior doctors because you are prepared to learn or you have learnt the basics. I don't expect them to know [everything], which is why we're consultants. (Examiner 10)

Drawing on CHAT, the interactions were explored between the identified sociocultural factors and the examiners’ expectations of student competence in the activity systems. Examiners’ expectations are tacit rules that guide individual examiners in their judgements of student competence. Since the examiners’ expectations were influenced by the cultural factor of their medical specialties in AS2, the examiners (subject) employed their expectations (rules), which could be different from the examiner community, to assess student competence in AS1 (Fig. 3). For example, examiners who are general practitioners might focus on a student’s communication skills as more important when compared to examiners from other specialty areas. Given the large number of examiners involved in this end-of-programme OSCE, different expectations contributed to the contradictions in AS1 especially when the examiners could not reach a consensus on the expected level of students’ competence during the briefing session.

The examiners’ experience and their expectations of student competence were reflected through the principle of historicity. The interactions of this historical factor are closely related to the community, division of labour and the object in AS1. There were two groups of examiners in the community: the experienced and the less-experienced examiners. The dominant voice from the experienced examiners often influenced the less-experienced examiners during the briefing session, for example, around expectations of student competence. Contradictions were created when the two groups of examiners were unable to reach a consensus of what should be expected in an OSCE station. Furthermore, the examiners’ social expectations of interns’ competence extend the analysis to help understand how their clinical roles in AS2 impacted on their expectations of the final-year medical student competence in AS1. The examiners expected that the final-year students should have already developed a solid foundation of medical knowledge and professional behaviour on which to build their clinical experience in the intern year.

The overall analysis of the examiners’ divergent expectations has illustrated the dynamic nature of the activity systems. The examiners’ divergent expectations are fundamentally associated with the examiners (subject), their expectations (rules) and the community in AS1. However, when exploring the element of division of labour based on the examiners’ experience of being OSCE examiners, the analysis reveals the interactions among the community, division of labour and object in AS1. Furthermore, the examiners’ medical specialties and the social influence (tacit rules) in AS2 also impacted on their expectations of students’ competence in progressing to the next stage of training in AS1 (Fig. 3). The interactions between the elements in AS1 and AS2 created contradictions in the examiners’ judgements of student competence in the end-of-programme OSCE. These contradictions resulted in different marking practices which influenced the consistency of the examiners’ judgements.

4. *Examiners’ idiosyncratic judgement practices*

Examiners’ idiosyncratic judgement practices were interrelated with their beliefs about the purpose of the end-of-programme OSCE, which were affected by their perceptions of the usefulness of the marking criteria. The examiners were required to provide their judgements on the station-specific marking criteria and a global impression of the student competence demonstrated in each station. The examiners typically applied two distinct practices to make their judgements. The first practice was that they provided their initial judgements of the marking standard (*very well; well; partially; poorly;* or*, not at all*) for each marking criterion (an individual score), and then they computed a summative judgement (an overall score) as their global impression of the demonstrated competence:

I find it easier if there's dot points that you can tick them off … that's how I come up with my mark. So there are some key points for every question … If they get none of the points, then they get marked poorly. If they get all of the ticks, then I tend to mark them exceptional. That's the way I do it. (Examiner 8)

The second practice was that the examiners made their initial judgements of a global impression of a student’s demonstrated competence, and then derived the individual marking standard for each marking criterion. Both practices were against the rules set by the medical school, that is, examiners should make a judgement of their global impression independently of each marking criterion scoring for standard-setting purposes. However, these rules were not explicitly stated on the marking criteria sheet.

Apart from the above two distinct marking practices, another example of idiosyncratic judgement practice was related to the examiners’ consideration of student professional behaviour such as their attitudes displayed throughout an OSCE station:

And this one other thing that will sway me and that’s their attitude towards the examination process …. If [the students] become a little bit aggressive or if they are not treating the process with respect, and they are half and half, then that’s more likely to tip me towards a fail. (Examiner 3)

Some examiners considered student attitudes demonstrated through their behaviour which were not specified in the medical school’s marking criteria, whereas some decided *not* to consider student attitudes as part of their judgements. These contradictory marking behaviours among the examiners could have led to the inconsistency of their judgements.

Applying CHAT facilitated critical reflection on the examiners’ idiosyncratic judgement practices which were associated with the interactions among the examiners, rules and the examiner community in the medical school OSCE activity system (AS1). The examiners’ habitual practice of using the marking criteria sheet without applying the explicit rules from the medical school built up contradictions within the community. These contradictions were related to the possible variations in translating the scores from each marking criterion to global, and vice versa, which might affect the objectivity of the examiners’ judgements. Moreover, experienced examiners who had broad experience in the clinical environment appeared to adopt a more global approach initially when they judged student competence. The examiners also articulated the reasons for their marking behaviour with reference to their experience in the clinical work environments AS2, which influenced their marking practice and created contradictions in AS1 when they adopted their own idiosyncratic judgements.

The examiners’ consideration of student attitudes displayed throughout an OSCE station was also influenced by the explicit rules in AS2 on an organisational level relating to professionalism outlined in the national interns’ training framework (Confederation of Postgraduate Medical Education Councils, 2012). If students showed aggressive and disrespectful behaviour when they did not know how to approach a challenging OSCE station, one examiner envisaged it was likely that the students would display similar attitudes during their internships in challenging situations, which would be an unacceptable standard of professional practice. The expectations of interns from the start and during their internships in AS2 could influence the examiners’ idiosyncratic judgements of student competence in the end-of-programme OSCE in AS1 (Fig. 3). Examiners’ idiosyncratic judgement practices pose the question of the objectivity of OSCEs even though examiners were provided with standardised marking criteria.

## Discussion

The findings contribute to addressing a gap in the literature on examiner behaviours, which has predominantly related to the desirable psychometric characteristics of OSCEs, or investigated examiners’ judgements from a cognitive perspective, through applying CHAT to explore the sociocultural complexities in examiners’ judgements and their implications for designing examiner training.

### Summary of key findings

CHAT was useful to guide a comprehensive analysis of the contradictions generated by the four identified factors: examiners’ contrasting beliefs about the purpose of the end-of-programme OSCE, their varying perceptions of usefulness of the OSCE marking criteria, their divergent sociocultural expectations of the final-year medical student competence, and their idiosyncratic judgement practices. CHAT facilitated the analysis of the impacts of the identified contradictions on a broader level taking into account the contextual background of this study such as the large cohort of final-year medical students (> 350) and the large number of examiners (> 100) involved in the end-of-programme OSCE. This broader analysis using CHAT extends the findings of the sociocultural factors that influence examiners’ judgements of student performance.

Using the terminology of CHAT, these differences are considered as contradictions among the elements such as the examiners (subject), marking criteria (tools) and making judgements of student competence (object). The guiding principle of multi-voicedness further facilitated a deeper understanding of how contradictions were created within the identified factors such as the level of guidance provided to students during an OSCE station in relation to the diverse examiners’ beliefs, and the examiners’ divergence from using the given marking criteria due to different perceptions of their usefulness and examiners’ experiences with interns in their clinical work. The guiding principle of historicity explored marking criteria changes over time in relation to the examiners’ perception of their usefulness. The same principle was also applied to investigate the length of time being OSCE examiners had on their diverse expectations of student competence in the end-of-programme OSCE.

### Comparison with existing literature

The analysis brings a novel approach to analyse OSCE practice and provides insight, in addition to the cognitive perspective (e.g., Gingerich et al., 2014; Yeates et al., 2013, 2019), on developing fit-for-purpose examiner training to enhance practices. Our findings of the key factors correspond to the recommendations for OSCEs such as “focus examiner training on conduct, behaviours and bias” (Boursicot et al., 2021, p. 61). CHAT could also be applied as an analytical framework to facilitate the understanding of the diversity among examiners, which helps raise awareness of the sociocultural factors that influence the consistency of examiners’ judgements of student performance and contribute to the design of fit-for-purpose training on examiner assessment practices.

The identification of the impactful contradictions among the elements on the organisational and system level help promote transformative change (Engeström, 2001), in this case, the development of fit-for-purpose examiner training. From the organisational perspective of exploring the two activity systems, the OSCE examiners (subject), who played a significant role only once a year to assess final-year students’ OSCE performance in a simulated setting in AS1, supervised and assessed interns’ performance with real-life patients regularly in AS2. The examiners’ roles in two different but interrelated contexts appear to create an impactful contradiction for them, requiring an adjustment of their expectations when assessing final-year students. This contradiction could also be due to the varying levels of satisfaction in working with interns and the different positions that the OSCE examiners held in the clinical context which could determine their level of involvement with the interns.

On a system level, the principle of multi-voicedness enables further exploration of another key social group in the community in AS1 (Table 1), that is, the simulated patients, in making judgements of student competence. Some examiners did indicate that they would consider the simulated patients’ point of view in making their judgements about some areas such as building rapport. Strengthening the partnerships with simulated patients (Brand & Dart, 2022) as part of the examiner training could also facilitate the examiners to adjust their expectations of student performance in the end-of-programme OSCE.

In addition, reflecting using the principle of historicity on the organisational and system level highlighted that an end-of-programme OSCE has traditionally been the final hurdle for medical students prior to commencing their internships across many medical schools in Australia in the absence of a national licensing examination. The end-of-programme OSCE has been conducted for more than 25 years in this medical school. An experienced OSCE examiner indicated that criterion-referenced marking was used back in 1995–1997 and this has contributed to the development of the examiner practice of ‘ticking off specific criteria’ to make judgements of student performance in OSCEs. This practice is the basis of the long-running debate on the validity of using item-based checklists to score student performance in OSCEs (Homer et al., 2020). Assessment rules at this medical school had changed to failing students on critical errors and returned to criterion-referenced marking when this study was conducted. These changes of the marking criteria created confusion among the experienced OSCE examiners.

Another finding that contributes to the literature is the impactful contradiction identified relating to the design of the marking criteria (tools), which did not meet the OSCE examiners’ expectations of facilitating the identification of students who were not competent to progress to the next stage of training. Given that the large examiner cohort consisted of experienced and less-experienced examiners, it is important for examiner training to offer an opportunity to develop mutual understanding of the purpose of the OSCE and assessment terminology used in the marking criteria (tools). Involving examiners in developing this understanding is a way to gain their support for making any necessary changes to the marking criteria (tools), which would help address the contradictions among the examiners (subject), marking criteria (tools), rules, and community in the activity system.

### Implications for assessment practice

To accommodate large cohorts of students and diversity of examiners who are also medical practitioners, it is important that examiner training offers an opportunity to develop two-way feedback between OSCE examiners and medical schools to align the expectations of student performance in the end-of-programme OSCE. The two-way feedback will also help medical schools develop a deeper understanding of the nuances of individual OSCE stations. The two-way communication between examiners and medical schools captures the essence of the activity systems in CHAT in that different elements relate to each other by continuously influencing and being influenced by others (Al-Ali, 2020). It is also important to strengthen partnerships with simulated patients in education and assessment (Brand & Dart, 2022) and develop mutual understanding of OSCE assessment terminology among the examiners. These initiatives could enable collaborations among examiners, simulated patients and the medical schools to rethink and realign the purpose, expectations and marking practices with the aim of addressing the contradictions at organisational and system levels.

### Implications for future research

CHAT has enabled the research team to explore a comprehensive set of dynamic factors and the contradictions created which impacted on the examiners’ judgements within the activity system of a medical school and across to the examiners’ clinical practice context. The CHAT methodology is recommended for future research in assessment exploring the relationships between the subjects, tools and rules within and across specific contexts. Future research should explore whether the identified sociocultural factors also influence examiners’ judgements in different OSCE contexts, for example, as a formative assessment to provide students with feedback to enhance their clinical skills development. Including stakeholders in the activity systems who have vested interest in the OSCE such as the medical school assessment leads, simulated patients and health services as the potential employers of the final-year medical students could further allow different voices to be considered.

### Strengths and limitations

This study was innovative in applying CHAT as an analytical framework to explore the sociocultural factors that influenced examiners’ judgement practices in the context of medical education. CHAT facilitates the analysis of a comprehensive set of dynamic elements from a systemic and multi-dimensional approach (Foot, 2014) within a single activity system (Engeström, 2014) (Fig. 1) and across interacting activity systems (Engeström, 2001) (Fig. 3). The use of the comparative method for themes saturation ensured the rigor of analysis of all the interview data in the context of our focused research question. Although the practices of assessing students using OSCEs vary widely depending on the context (e.g., size of the student and examiner cohorts, number and duration of the stations, and the comprehensiveness of examiner training), the OSCE setting in this study is likely to occur at other institutions with a large cohort of students and time pressure on examiners who are also clinicians. A limitation of this study is that only the OSCE examiners were interviewed. They were on a continuum from teaching at all levels, to specific clinical specialty rotations, to assessment only and the demarcations were unclear. Nevertheless, the findings help develop a deeper understanding of the complexity of the sociocultural factors from the examiners’ perspective that influenced their judgements of student OSCE performance and suggest practical recommendations for examiner training initiatives.

## Conclusions

This study identified four key sociocultural factors: examiners’ beliefs, perceptions, expectations and idiosyncratic judgement practices that influence their judgements of students’ competence in progressing to the next stage of clinical training in the end-of-programme OSCE. These factors could impact the perceived objectivity of the OSCEs. Applying CHAT as an analytical framework brings a novel approach to analyse OSCE practice and raises awareness of the sociocultural factors that influence the consistency of examiners’ judgements. The guiding principles of multi-voicedness and historicity further facilitated the understanding of contradictions created within the identified factors such as the level of guidance provided in relation to the examiners’ beliefs, and changes of marking criteria over time associated with the examiners’ perception of their usefulness. The analysis provides insight, in addition to the cognitive perspective, on developing fit-for-purpose examiner training to enhance assessment practices for the benefits of our students, examiners, and patients.

## Supplementary Information

Below is the link to the electronic supplementary material.

Supplementary file1 (DOCX 13 kb)
