# Supplementary material for: Using cultural historical activity theory to reflect on the sociocultural complexities in OSCE examiners’ judgements
Source: Adv Health Sci Educ Theory Pract. 2022 Aug 9;28(1):27–46. doi: 10.1007/s10459-022-10139-1 (PMC9992227; doi:10.1007/s10459-022-10139-1)
Supplement: Supplementary file 1 — Supplementary file1 (DOCX 13 kb) [file 10459_2022_10139_MOESM1_ESM.docx]

**Online Appendix 1**. Interview questions with the OSCE examiners.

The interview questions asked that were relevant to this study are as follows:

- With your experience as an OSCE examiner, what do you see as the major challenges in making judgements of student performance in clinical examinations/OSCEs?
- How do you interpret ‘leniency’ and ‘stringency’?
- Do you perceive yourself as a lenient or as a stringent marker? Why?
- What are the factors that influence your judgement as a lenient or as a stringent examiner?
- What are the factors that influence your judgement of medical student performance in the OSCE?
